# ﻿Three new species of *Teunia* (Cryptococcaceae, Tremellales) identified through phenotypic and phylogenetic analyses

**DOI:** 10.3897/mycokeys.105.120534

**Published:** 2024-05-15

**Authors:** Qi-Chao Guo, Shan Liu, Ya-Zhuo Qiao, Feng-Li Hui

**Affiliations:** 1 School of Life Science and Agricultural Engineering, Nanyang Normal University, Nanyang 473061, China Nanyang Normal University Nanyang China; 2 Research Center of Henan Provincial Agricultural Biomass Resource Engineering and Technology, Nanyang Normal University, Nanyang 473061, China Nanyang Normal University Nanyang China

**Keywords:** Basidiomycota, fungal diversity, new species, plant, taxonomy

## Abstract

*Teunia*, belonging to the family Cryptococcaceae of the order Tremellales, is a genus of plant-inhabiting fungi distributed across the globe. Its members form associations with different plant parts, including flowers, fruits, leaves, seeds, and twigs. Recent efforts have aimed to explore the diversity of *Teunia* in China, however, many geographical regions have not yet been explored. In this study, we included results of five *Teunia* yeast strains that were isolated from plant materials collected in Fujian, Guizhou and Henan provinces, with descriptions, illustrations, and phylogenetic analyses of three new species: *T.acericola*, *T.mussaendrae* isolated from leaf surfaces in Fujian, Guizhou and Henan Provinces, and *T.qingyuanensis* obtained from rotting wood in Fujian Province.

## ﻿Introduction

*Teunia* is a recently established genus by Li et al. (2020), based on the phylogenetic analysis of a seven-gene dataset consisting of SSU rRNA, D1/D2 LSU rRNA domain, ITS region, RPB1, RPB2, TEF1-α, and CYTB. This analysis revealed a well-supported clade encompassing *Cryptococcuscuniculi* K.S. Shin & Y.H. Park, *Fonsecazymatronadorensis* Yurkov (= *Cryptococcustronadorensis* V. de García, Zalar, Brizzio, Gunde-Cim & Van Broock), *Fonsecazymabetulae* Yurkov, Kachalkin & Boekhout (= *Kwoniellabetulae* K. Sylvester, Q.M. Wang & Hittinger), along with three new species *T.globosa* Q.M. Wang, F.Y. Bai & A.H. Li, *T.helanensis* Q.M. Wang, F.Y. Bai & A.H. Li, and *T.korlaensis* Q.M. Wang, F.Y. Bai & A.H. Li that was designated as the type species of the genus (Li et al. 2020). Since then, the increasing accessibility of sequencing services and a large quantity of available molecular data have led to a rapid expansion in the knowledge of the genus, and seven new species have been described: *T.rosae* Q.M. Wang, A.H. Li, G.S. Wang & Wangmu, *T.rudbeckiae* Q.M. Wang, A.H. Li, G.S. Wang & Wangmu (Wang et al. 2020), *T.siamensis* Khanam & Limtong (Khunnamwong et al. 2020), *T.lichenophila* Kachalkin, M.A. Tomashevskaya & T.A. Pankrato (Crous et al. 2021), *T.nitrariae* X.Z. Liu, F.Y. Bai & X.Y. Wei (Wei et al. 2022), and *T.virginiahalliae* Y.P. Tan & G.S. Pegg (Tan and Pegg 2023). In the case of *T.virginiahalliae*, which has been proposed based only on the ITS sequence, a representative reference culture has not been deposited in a culture collection, which hampers further studies on this species.

Until now, 12 species have been accepted in *Teunia* (www.indexfungorum.org/; www.mycobank.org). They all share cream to yellow-colored colonies, polar budding, non-fermentative nature, and inability to form pseudohyphae, hyphae, and ballistoconidia (Li et al. 2020). The members of *Teunia* have been found in diverse habitats and are frequently isolated as epiphytes from flowers (Wang et al. 2020), leaves (Sylvester al. 2015; Li et al. 2020), and tree barks (Sylvester et al. 2015), *T.lichenophila* was isolated as endophyte from *Cladoniarangiferina* and *C.stellaris* (Crous et al. 2021). Species of *Teunia* have also been isolated from soil (Khunnamwong et al. 2020; Li et al. 2020), barley from wild rabbit feces (Shin et al. 2006) and glacial biomes (de Garcia et al. 2012). Furthermore, it is hypothesized that an excess of 30 undescribed or erroneously identified strains may represent an additional 20 *Teunia* species (Wang et al. 2020). These potential members originate from various diverse substrates, including plant materials such as flowers (Herzberg et al. 2002; Mittelbach et al. 2015), floral nectars (Alvarez-Pérez and Herrera 2013), seeds (Fernández et al. 2012), fruits, leaves, and twigs. Others have been collected from soil (Takashima et al. 2012; Yurkov et al. 2016), coastal seawater, and extreme acidic environments (Gadanho et al. 2006). Taken together, these previous findings could be an indication that the habitat of these fungi is different plant parts.

Currently, half of the accepted species in *Teunia* were described from China, *T.globosa*, *T.helanensi*, *T.korlaensis* (Li et al. 2020), *T.rosae*, *T.rudbeckiae* (Wang et al. 2020), and *T.nitrariae* (Wei et al. 2022). However, these species have been collected from limited geographical ranges, and it is hoped that broader field investigations will reveal additional members of the genus.

During our investigation, we isolated five strains of *Teunia* from various substrates across different regions of China. Our phylogenetic analyses and examination of phenotypic features determined that the isolates represent three new species. The objective of this paper is to describe these species with morphological and molecular characters and contribute to knowledge of the diversity of *Teunia* in China.

## ﻿Materials and methods

### ﻿Sample collection and yeast isolation

Materials were collected from the Fujian, Guizhou, and Henan Provinces of China. One of the yeast strains was isolated from rotting wood through the enrichment method described by Shi et al. (2021). Four additional strains were harvested from leaf surfaces using the improved ballistospore-fall method described by Nakase and Takashima (1993). Based on this method, fresh leaves were cut into small pieces and adhered with a thin layer of petroleum jelly to the inner lid of a Petri dish containing yeast extract-malt extract (YM) agar (0.3% yeast extract, 0.3% malt extract, 0.5% peptone, 1% glucose, and 2% agar). The mixture was supplemented with 0.01% chloramphenicol to avoid bacterial growth. Plates were incubated at 20 °C and monitored daily for colony formation. Selected colonies were streaked onto separate YM agar plates for purification. Following purification, strains were suspended in YM broth supplemented with 20% (v/v) glycerol and stored at –80 °C for future use. Cultures of all obtained isolates were preserved at the Microbiology Lab, Nanyang Normal University, Henan, China.

### ﻿Phenotypic characterization

Morphological, physiological, and biochemical analyses were performed according to the standard methods described by Kurtzman et al. (2011). To examine the inducibility of the sexual state in each isolate, single or double strains were mixed on corn meal agar (CMA), potato dextrose agar (PDA), and V8 agar (10% V8 juice, 2% agar) at 20 °C for up to 8 weeks (Wang et al. 2020). Glucose fermentation was tested in a liquid medium with Durham fermentation tubes. Carbon and nitrogen assimilation capabilities were examined in a liquid medium, with starved inoculum used for nitrogen testing (Kurtzman et al. 2011). Growth at various temperatures (15, 20, 25, 30, 35, and 37 °C) was assessed through cultivation on YM agar plates. Cell morphology was examined with LEICA DM2500 cameras (LECIA Co, Wetzlar, Germany) and LASV4.13 software. All proposed names and descriptions were deposited in the MycoBank database (http://www.mycobank.org; 8 February 2024).

### ﻿DNA extraction, PCR amplification, and sequencing

Genomic DNA was extracted from each strain using the Ezup Column Yeast Genomic DNA Purification Kit, according to the manufacturer’s instructions (Sangon Biotech Co., Shanghai, China). The ITS region, D1/D2 domain of the LSU rRNA, and a partial segment RPB1 were amplified with primers ITS1/ITS4 (White et al. 1990), NL1/NL4 (Kurtzman and Robnett 1998), and RPB1-Af and RPB1-Cr (Kurtzman and Robnett 2003), respectively. Amplifications were performed in a 25 µL reaction-volume tube containing 9.5 µL ddH_2_O, 12.5 µL Taq 2X PCR Master Mix with blue dye (Sangon Biotech Co., Shanghai, China), 1 µL DNA template, and 1 µL of each primer. The ITS region and D1/D2 domain were amplified with an initial denaturation step of 2 min at 95 °C, followed by 35 cycles of 30 s at 95 °C, 30 s at 51 °C, 40 s at 72 °C, and a final extension of 10 min at 72 °C (Toome et al. 2013). Amplification of the partial RPB1 gene was conducted using a touchdown PCR protocol as described by Wang et al. (2014). PCR products were then purified and sequenced by Sangon Biotech Co., Ltd (Shanghai, China) using the same primers. The identity and accuracy of each sequence were determined by comparison to sequences in GenBank. Assembly was performed with BioEdit v.7.1.3.0 (Hall 1999). All newly generated sequences were deposited in the GenBank database (https://www.ncbi.nlm.nih.gov/genbank/).

### ﻿Phylogenetic analysis

Phylogenetic analyses were conducted based on LSU sequences alone and a combination of the ITS, LSU, and RPB1 dataset. *Cryptococcusamylolentus* CBS 6039^T^ and *Cryptococcusneoformans* CBS 8710^T^ were designated as outgroups (Crous et al. 2021). Individual loci sequences were aligned using MAFFT v.7.110 (Katoh and Standley 2013) under the G-INI-I option. Poorly aligned regions were removed and adjusted manually using MEGA v.11 (Tamura et al. 2021). Aligned sequences of the different loci were concatenated with Phylosuit v.1.2.2 (Zhang et al. 2020).

Maximum likelihood (ML) analysis was conducted using RAxML v.8.2.3 with the GTRGAMMA model (Stamatakis 2014). Node ML bootstrap values (MLBS) were evaluated using 1,000 rapid replicates. The Best-fit evolution model for Bayesian inference (BI) was determined with ModelFinder (Kalyaanamoorthy et al. 2017). BI analysis was performed using MrBayes v.3.2.7a (Ronquist et al. 2012) through the CIPRES Science Gateway. Six simultaneous Markov chains were run for 50 million generations, with trees sampled every 1,000^th^ generation. The first 25% of trees were discarded, representing the burn-in phase. Remaining trees were used to calculate the Bayesian posterior probabilities (BPP) of each clade. Trees were examined using FigTree v.1.4.3 (Andrew 2016). Branches exhibiting MLBS values ≥50% and BPP values ≥0.95 were shown at the nodes.

## ﻿Results

### ﻿Molecular phylogeny

A total of five yeast strains preliminarily identified as *Teunia* were studied further (Table 1). Besides the newly generated sequences, additional related sequences were also downloaded from GenBank (Table 2) for inclusion in the phylogenetic analyses.

**Table 1.** Yeast strains and origins investigated in this study.

Strain	Source	Location
***Teuniaacericola* Y.Z. Qiao & F.L. Hui**		
NYNU 2111141^T^	Leaf of *Acerpalmatum*	Baotianman Nature Reserve, Neixiang County, Henan Province, China
NYNU 2111157	Leaf of *Rhuschinensis*	Baotianman Nature Reserve, Neixiang County, Henan Province, China
***Teuniaqingyuanensis* Y.Z. Qiao & F.L. Hui**		
NYNU 22475^T^	Rotting wood	Qingyuan Mountain, Quanzhou City, Fujian Province, China
***Teuniamussaendrae* Y.Z. Qiao & F.L. Hui**		
NYNU 23232^T^	Leaf of *Mussaendapubescens*	Sifangjing Village, Pingtang County, Guizhou Province, China
NYNU 23257	Leaf of *Viburnum* sp.	Sifangjing Village, Pingtang County, Guizhou Province, China

**Table 2.** Species name, strain/clone numbers, and GenBank accession numbers included in phylogenetic analyses. Entries in bold represent newly generated materials.

Species name	Strain/clone number	GenBank accession numbers		
		ITS	LSU D1/D2	RPB1
*Cryptococcusamylolentus*	CBS 6039^T^	NR_111372	KY106966	KF036342
*Cryptococcusneoformans*	CBS 8710^T^	NR_171785	NG_058766	KF036472
*Kwoniellabestiolae*	CBS 10118^T^	NR_111373	NG_042482	KF036351
*Kwonielladejecticola*	CBS 10117^T^	NR_111374	NG_042483	KF036362
*Kwonielladendrophila*	CBS 6074^T^	NR_073257	NG_058326	KF036320
*Kwoniellaendophytica*	CBS 15359^T^	MH237945	MH237945	LS992197
*Kwoniellaheveanensis*	CBS 569^T^	NR_073210	AF075467	FJ534921
*Kwoniellamangrovensis*	CBS 8507^T^	NR_073332	AF444742	KF036498
*Kwoniellaovata*	CGMCC 2.3439^T^	NR_174734	MK050289	MK849160
*Kwoniellapini*	CBS 10737^T^	NR_111269	KY108203	KF036395
*Kwoniellashivajii*	CBS 11374^T^	NR_165977	NG_042515	KF036401
***Teuniaacericola***	**NYNU 2111141^T^**	**OM017172**	**OM017170**	**PP236726**
***Teuniaacericola***	**NYNU 2111157**	**PP239073**	**PP239062**	**PP236727**
*Teuniaacericola*	BI226	–	EU678944	–
*Teuniaacericola*	HB31-3	–	KJ507251	–
*Teuniaacericola*	MUCC1912	–	LC715712	–
*Teuniaacericola*	MUCC2071	–	LC715721	–
*Teuniaacericola*	F3-5	–	AB618905	–
*Teuniabetulae*	NRRL Y-63732^T^	KM384102	KM408130	–
*Teuniacuniculi*	CBS 10309^T^	NR_137887	KY106982	MN014082
*Teuniaglobosa*	CGMCC 2.5648^T^	NR_174733	MK050288	MK849235
*Teuniahelanensis*	CGMCC 2.4450^T^	NR_174732	MK050287	MK849208
*Teuniakorlaensis*	CGMCC 2.3835^T^	NR_174731	MK050286	MK849194
*Teunialichenophila*	CBS 16716^T^	MN128421	MN128421	HG992858
***Teuniamussaendrae***	**NYNU 23232^T^**	**OQ851888**	**OQ851887**	**PP236729**
***Teuniamussaendrae***	**NYNU 23257**	**PP239074**	**PP239072**	**PP236730**
*Teunianitrariae*	CGMCC 2.6797^T^	OM417183	OM417183	–
***Teuniaqingyuanensis***	**NYNU 22475^T^**	**OP269841**	**OP269842**	**PP236728**
*Teuniarosae*	CGMCC 2.5830^T^	MK942578	MK942560	MT268696
*Teuniarudbeckiae*	CGMCC 2.5840^T^	MK942577	MK9425595	MT268698
*Teuniasiamensis*	DMKU-XD44^T^	LC440108	LC420623	–
*Teuniatronadorensis*	DSM 26994^T^	MF959620	MF959620	–
*Teuniavirginiahalliae*	BRIP 64084e^T^	OR660683	–	–

**^T,^** type strain. Species obtained in this study are in bold.

**Figure 1.**
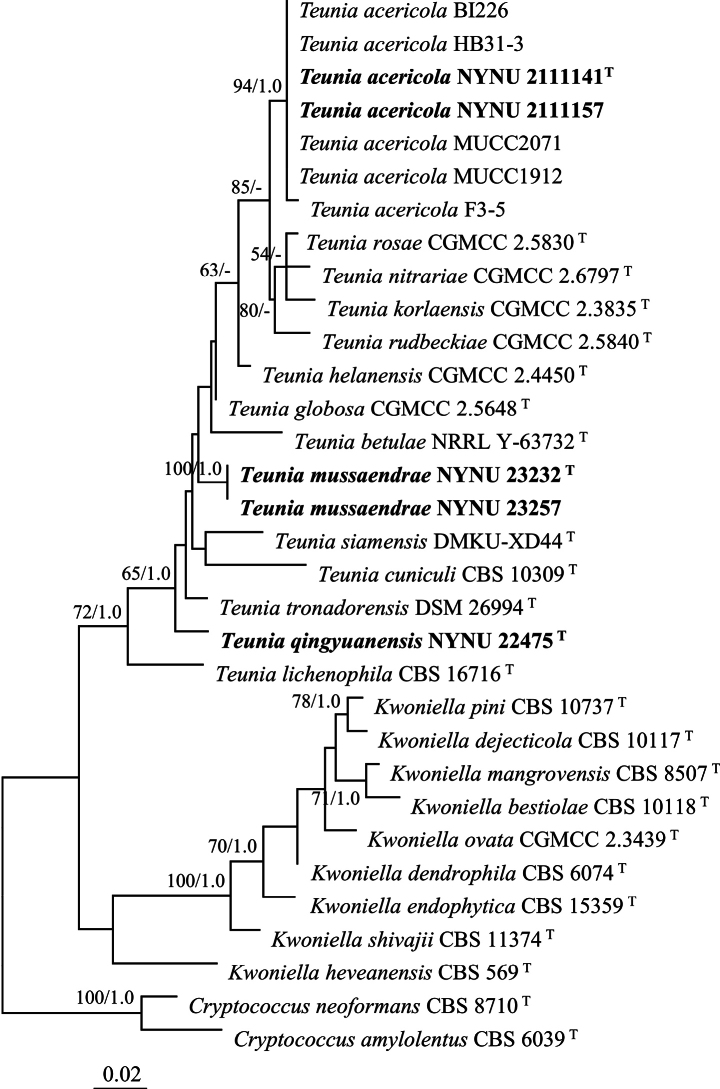
Maximum likelihood phylogenetic tree of *Teunia* generated from the LSU sequence data. The tree is rooted with *Cryptococcusamylolentus* CBS 6039^T^ and *Cryptococcusneoformans* CBS 8710^T^. Bootstrap values (MLBS ≥ 50% and BPP ≥ 0.95) are displayed near branches. Type strain sequences are marked with (T).

The LSU dataset consisted of 32 sequences representing 25 species. The aligned set had a length of 603 characters, of which 480 were constant, 34 were variable and parsimony-uninformative, and 89 were parsimony-informative. The BI yielded a topology similar to the ML analysis, with an average standard deviation of split frequencies equal to 0.009938. In the LSU based phylogenetic tree (Fig. 1), five newly isolated strains formed three distinct and well-supported lineages that are distant from other *Teunia* species. Since *T.virginiahalliae* only has ITS sequence data, the phylogenetic analysis based on the ITS dataset was also performed. The phylogenetic tree (Suppl. material 1) recovered 12 known species of *Teunia*, while the newly isolated strains formed three independent lineages as in the phylogeny inferred from the LSU dataset.

**Figure 2.**
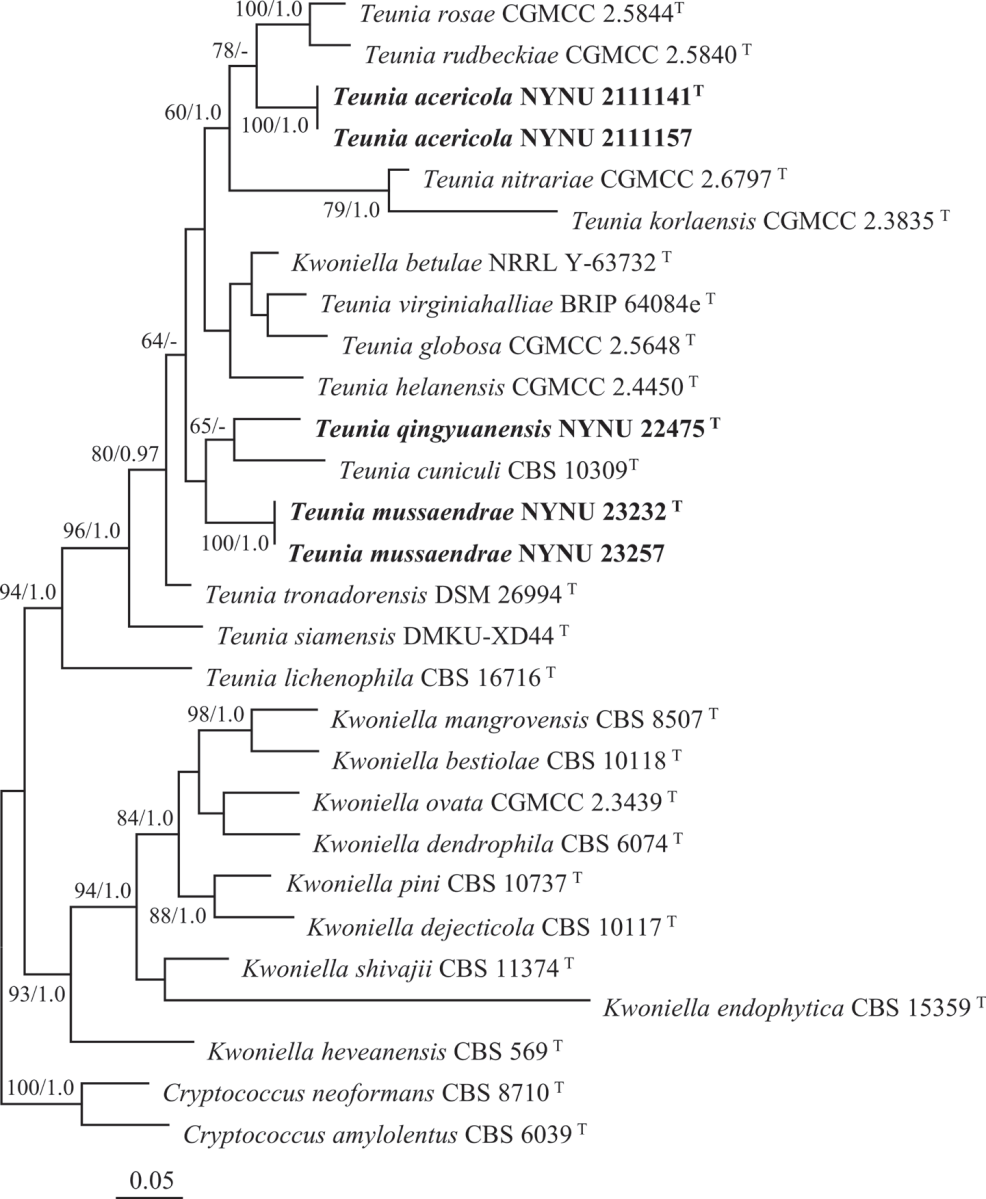
Maximum likelihood phylogenetic tree of *Teunia* generated from the combined ITS, LSU, and RPB1 sequence data. The tree is rooted with *Cryptococcusamylolentus* CBS 6039^T^ and *Cryptococcusneoformans* CBS 8710^T^. Bootstrap values (MLBS ≥ 50% and BPP ≥ 0.95) are displayed near branches. Type strain sequences are marked with (T).

The combined ITS, LSU, and RPB1 dataset encompassed sequences from 28 yeast strains representing 26 species. Including gaps, the dataset had an aligned length of 1,978 characters (549, 603, and 826 characters for ITS, LSU, and RPB1, respectively), of which 873 were constant, 381 were variable and parsimony-uninformative, and 724 were parsimony-informative. The best-fit model of the combined dataset for BI analysis was determined to be GTR+I+G, with equal nucleotide frequencies. The BI yielded a topology similar to the ML analysis, with an average standard deviation of split frequencies equal to 0.009550. The ITS, LSU, and RPB1 based phylogenetic tree (Fig. 2) produced a topology similar to that generated by the LSU based phylogenetic tree, and further confirmed the groupings of the three new species within *Teunia*.

Strains NYNU 2111141 and NYNU 2111157 were isolated from different leaves, but possess identical D1/D2 and ITS sequences. Both phylogenetic trees (Figs 1, 2) revealed that these two strains clustered with *T.korlaensis*, *T.nitrariae*, *T.rosae*, and *T.rudbeckiae*, with variations of eight to10 nt (~1.3–1.7%) substitutions in the D1/D2 domain and more than 19 nt (~3.7%) mismatches in the ITS region. This suggests that the strains represent a novel *Teunia* species. A search of GenBank for entries associated with our test isolates unveiled the sequences of four unpublished strains: ‘*Cryptococcus*’ sp. BI226 (EU678944), ‘*Kwoniella*’ sp. HB31-3 (KJ507251), *Teunia* sp. MUCC1912 (LC715712), and *Teunia* sp. MUCC2071 (LC715721), along with an uncultured fungus clone F3-5 (AB618905) (Fig. 1). These sequences exhibit highly similar D1/D2 domain (0–2 nt differences) when compared with NYNU 2111141 and NYNU 2111157. This suggests they may all belong to the same novel species, for which we propose the name *Teuniaacericola* sp. nov.

Isolated from rotting wood, strain NYNU 22475 formed a branch distant from the other *Teunia* species in the D1/D2 phylogenetic tree (Fig. 1). However, the tree based on the combined ITS, LSU, and RPB1 dataset weakly supported a cluster with *T.cuniculi* CBS 10309 (Fig. 2). The two strains differed by 16 nt (~2.9%) substitutions in the D1/D2 domain and 22 nt (~4.3%) mismatches in the ITS region, suggesting they are closely related but do not belong to the same species. Taken together, these findings indicate that NYNU 22475 represents a novel *Teunia* species, for which we propose the name *Teuniaqingyuanensis* sp. nov.

Finally, isolated from separate leaves, strains NYNU 23232 and NYNU 23257 were found to possess identical sequences and formed an independent single-species lineage in the D1/D2 phylogenetic tree (Fig. 1). The ITS, LSU, and RPB1 combined tree presented a non-supported cluster with *T.cuniculi* and the newly discovered *T.qingyuanensis* sp. nov. (Fig. 2). BLASTn searches using D1/D2 sequences indicated that novel strains were most closely related to *T.globosa*, with variations of eight nt (~1.4%) substitutions in the D1/D2 domain and 28 nt (~5%) mismatches in the ITS region. Based on the ITS region, *T.virginiahalliae* represented the closest relative, differing by 19 nt (~3.4%) substitutions. The D1/D2 sequence of *T.virginiahalliae* was not available for comparison. Thus, it was determined that NYNU 23232 and NYNU 23257 represent a novel *Teunia* species, for which we propose the name *Teuniamussaendrae* sp. nov.

### ﻿Taxonomy

#### ﻿ Teunia acericola

Taxon classificationFungiTremellalesCryptococcaceae

Y.Z. Qiao & F.L. Hui sp. nov.

85E0A549-64E0-57A4-A733-CC530479889A

 852101

Fig. 3A

##### Etymology.

Referring to *Acer*, the genus of the plant where the type strain was isolated.

##### Typus.

China. Henan Prov. Neixiang Co., Baotianman Nature Reserve; in the phylloplane of *Acerpalmatum*; Nov 2021; R.R.Jia & W.T.Hu; NYNU 2111141 (holotype CICC 33544^T^, culture ex-type JCM 35732; GenBank Nos: OM017172, OM017170, PP236726).

**Figure 3.**
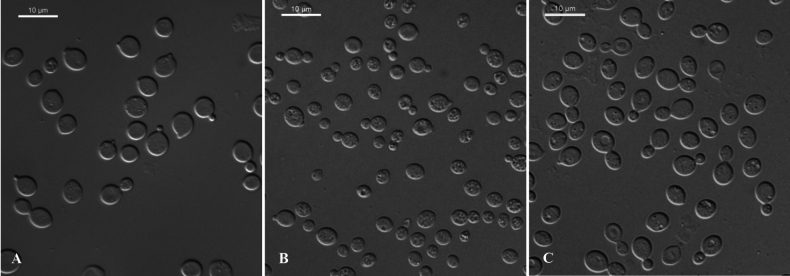
Vegetative cells of **A***T.acericola* sp. nov. NYNU 2111141^T^**B***T.qingyuanensis* sp. nov. NYNU 22475^T^ and **C***T.mussaendrae* sp. nov. NYNU 23232^T^, following 7 days of growth in YM broth at 20 °C. Scale bars: 10 μm.

##### Description.

On YM agar after seven days at 20 °C, the streak culture was cream, mucoid, smooth, with entire margin. After seven days in YM broth at 20 °C, single cells were globose to ovoid, 2.5–5.5 × 4–6 μm, budding polar. After one month at 20 °C, sediment was present. In Dalmau plate culture on CMA, no hyphae or pseudohyphae were formed. Sexual structures were not observed in any of the strains or when strains are paired on PDA, CMA or V8 agar. Glucose fermentation was absent. Glucose, inulin, sucrose, raffinose, melibiose, galactose, lactose, trehalose, maltose, melezitose, cellobiose, salicin, L-sorbose, L-rhamnose, D-xylose, L-arabinose, D-arabinose (weak), 5-keto-D-gluconate, D-ribose, ethanol (weak), glycerol, ribitol, galactitol, D-mannitol, D-glucitol, myo-inositol, DL-lactate, succinate, D-gluconate, D-glucosamine (weak), 2-keto-D-gluconate, D-glucuronate, and glucono-1,5-lactone were assimilated as carbon sources; methanol, erythritol, and N-acetyl-D-glucosamine were not assimilated. Ethylamine and L-lysine were assimilated as nitrogen sources, nitrate, nitrite, and cadaverine were not assimilated. Maximum growth temperature was 35 °C. Growth in vitamin-free medium was negative. Growth on 50% (w/w) glucose-yeast extract agar was negative. Starch-like substances were not produced. Urease activity and Diazonium Blue B reaction were positive.

##### Additional strain examined.

China. Henan Prov. Neixiang Co., Baotianman Nature Reserve; in the phylloplane of *Rhuschinensis*; Nov 2021; R.R.Jia & W.T.Hu; NYNU 2111157 (GenBank No: PP239073, PP239062, PP236727).

##### Note.

In the molecular analysis (Figs 1, 2), *T.acericola* sp. nov. was clustered with *T.korlaensis*, *T.nitrariae*, *T.rosae*, and *T.rudbeckiae. T.acericola* sp. nov. can be differentiated from those four species by its ability to assimilate raffinose and its growth capacity at 35 °C.

#### ﻿ Teunia qingyuanensis

Taxon classificationFungiTremellalesCryptococcaceae

Y.Z. Qiao & F.L. Hui sp. nov.

8F6ACFCF-5175-57CF-B1CD-F6FA96CA0432

 852102

Fig. 3B

##### Etymology.

Referring to the locality, Qingyuan Mountain, where the type strain was collected.

##### Typus.

China. Fujian Prov. Quanzhou City, Qingyuan Mountain; in rotting wood; Mar 2022; W.T.Hu & S.B.Chu; NYNU 22475 (holotype GDMCC 2.294^T^, culture ex-type PYCC 9929; GenBank Nos: OP269841, OP269842, PP236728).

##### Description.

On YM agar after seven days at 20 °C, the streak culture was cream, mucoid and smooth, with an entire margin. After seven days in YM broth at 20 °C, single cells were ovoid to ellipsoidal, 3–7 × 4–7.5 μm, budding polar. After one month at 20 °C, sediment was present. In Dalmau plate culture on CMA, no hyphae or pseudohyphae were formed. Sexual structures were not observed on PDA, CMA or V8 agar. Glucose fermentation was absent. Glucose, inulin, sucrose, raffinose, melibiose, galactose, lactose, trehalose, maltose, melezitose, cellobiose, salicin, L-sorbose (weak), L-rhamnose, D-xylose, L-arabinose, D-arabinose, 5-keto-D-gluconate, D-ribose, ethanol, glycerol, ribitol, galactitol, D-mannitol, D-glucitol, myo-inositol, DL-lactate, succinate, D-gluconate, 2-keto-D-gluconate, D-glucuronate, and glucono-1,5-lactone were assimilated as carbon sources; methanol, erythritol, and D-glucosamine were not assimilated. Ethylamine and L-lysine were assimilated as nitrogen sources; nitrate, nitrite, and cadaverine were not assimilated. Maximum growth temperature was 30 °C. Growth in vitamin-free medium was positive. Growth on 50% (w/w) glucose-yeast extract agar was negative. Starch-like substances were not produced. Urease activity and Diazonium Blue B reaction were positive.

##### Note.

In the molecular analysis (Fig. 2), *T.qingyuanensis* sp. nov. formed a distinct clade together with *T.cuniculi*. They can be differentiated by the ability of *T.qingyuanensis* sp. nov. to grow in vitamin-free medium and to assimilate raffinose, melibiose, and DL-Lactate.

#### ﻿ Teunia mussaendrae

Taxon classificationFungiTremellalesCryptococcaceae

Y.Z. Qiao & F.L. Hui sp. nov.

6DAACF92-F702-5E2B-942B-35314CD9385C

 852103

Fig. 3C

##### Etymology.

Referring to *Mussaenda*, the genus of the plant where the type strain was isolated.

##### Typus.

China. Guizhou Prov. Pingtang Co., Sifangjing Vil.; in the phylloplane of *Mussaendapubescens*; Feb 2023; D.Lu; NYNU 23232 (holotype CICC 33594^T^, culture ex-type PYCC 9974; GenBank Nos OQ851888, OQ851887, PP236729).

##### Description.

On YM agar after seven days at 20 °C, the streak culture was yellowish-cream, mucoid and smooth, entire margin. After seven days in YM broth at 20 °C, cells isolated were globose to ovoid, 3.5–5 × 4.5–6 μm, budding polar. After one month at 20 °C, a ring and sediment was present. In Dalmau plate culture on CMA, no hyphae or pseudohyphae were formed. Sexual structures were not observed in any of the strains or when strains were paired on PDA, CMA or V8 agar. Glucose fermentation was absent. Glucose, inulin, sucrose, galactose, lactose, trehalose, maltose, melezitose, cellobiose, salicin, L-sorbose, L-rhamnose, D-xylose, L-arabinose, D-arabinose, 5-keto-D-gluconate, D-ribose, ethanol (weak), glycerol (weak), ribitol, galactitol, D-mannitol, D-glucitol, myo-inositol, DL-lactate, succinate, D-gluconate, D-glucosamine (weak), 2-keto-D-gluconate, D-glucuronate, and glucono-1,5-lactone were assimilated as carbon sources; raffinose, melibiose, methanol, erythritol, and N-acetyl-D-glucosamine were not assimilated. Ethylamine (delayed), L-lysine, and cadaverine (delayed) were assimilated as nitrogen sources; nitrate and nitrite were not assimilated. Maximum growth temperature was 25 °C. Starch-like substances were not produced. Urease activity and Diazonium Blue B reaction were positive.

##### Additional strain examined.

China. Guizhou Prov. Pingtang Co., Sifangjing Vil.; in the phylloplane of *Viburnum* sp.; Feb 2023; D.Lu; NYNU 23257 (GenBank Nos: PP239074, PP239072, PP236730).

##### Note.

Based on the D1/D2 sequences, *T.mussaendrae* sp. nov. was most closely related to *T.globosa*. It can be differentiated from *T.globosa* by the ability to assimilate L-sorbose, L-arabinose, D-arabinose, ribitol, galactitol, D-glucitol, and D-gluconate. Additionally, *T.mussaendrae* sp. nov. can grow in vitamin-free medium at 25 °C, while *T.globosa* cannot.

## ﻿Discussion

Our study confirms that three species with similar colors, colony morphology, and cell shapes, can be distinguished from previously described species using the polyphasic approach recommended by Li et al. (2020) and Wang et al. (2020). In this case we use physiological and biochemical characters as well as morphological and phylogenetic ones.

The genus *Teunia* is widely distributed in China, but knowledge about it is still in its infancy. The six species previously reported, come mainly from the northern regions (Li et al. 2020; Wang et al. 2020; Wei et al. 2022). The exploration of new territories, such as that carried out in the provinces of Fujian, Guizhou and Henan, is necessary to have a more exact knowledge of their distribution and ecology. The results presented in this paper increase the total number of *Teunia* species from six to nine.

Furthermore, four unpublished strains, BI226 from Brazil, HB31-3 from South Korea, MUCC1912 and MUCC2071 from Japan, as well as an uncultured fungus clone F3-5 from Japan, are conspecific with *T.acerica* sp. nov. These observations suggest that this species can have a wide distribution area. Therefore, a broader taxon sampling effort, coupled with molecular, phenotypic, physiological and biochemical data, is needed to fully understand the species diversity of *Torula* in the world.

The species of *Teunia* are frequently isolated as epiphytes from different parts of herbaceous plants, more rarely from tree barks or lichens; in this case, we isolated five yeast strains, which led to the discovery of three new species: *T.acericola* sp. nov., *T.mussaendrae* sp. nov. isolated from leaf surfaces, and *T.qingyuanensis* sp. nov. from rotting wood. We have found no previous reports of the presence of *Teunia* in rotting wood in China, hence our study is the first to report the presence of *Teunia* in rotten wood in China.

*Teuniakorlaensis* and *T.nitrariae* are versatile extremophilic species that have been frequently found in plants inhabiting dry and alkaline environments (Wei et al. 2022), implying that these species may help plants survive in dry areas. We also isolated four strains of two novel *Teunia* species - *T.acericola* sp. nov. and *T.mussaendrae* sp. nov. - from plant leaves, and it is possible that these species provide similar ecological functions’ benefits to their hosts as do *T.korlaensis* and *T.nitrariae*.

## Supplementary Material

XML Treatment for Teunia acericola

XML Treatment for Teunia qingyuanensis

XML Treatment for Teunia mussaendrae

## ﻿Additional information

### Author ORCIDs

Qi-Chao Guo https://orcid.org/0009-0002-9245-479X

Shan Liu https://orcid.org/0009-0003-2845-1495

Ya-Zhuo Qiao https://orcid.org/0009-0000-9074-2443

Feng-Li Hui https://orcid.org/0000-0001-7928-3055

### Data availability

All of the data that support the findings of this study are available in the main text or Supplementary Information.

## Supplementary materials

Supplementary material 1 Supplementary data Data type pdf Explanation note **fig. S1.** Maximum likelihood phylogenetic tree of *Teunia* generated from the ITS sequence data. https://binary.pensoft.net/file/1049626This dataset is made available under the Open Database License (http://opendatacommons.org/licenses/odbl/1.0/). The Open Database License (ODbL) is a license agreement intended to allow users to freely share, modify, and use this Dataset while maintaining this same freedom for others, provided that the original source and author(s) are credited.Qi-Chao Guo, Shan Liu, Ya-Zhuo Qiao, Feng-Li Hui
