# Supplementary material for: ﻿Three new species of Teunia (Cryptococcaceae, Tremellales) identified through phenotypic and phylogenetic analyses
Source: MycoKeys. 2024 May 15;105:139–53. doi: 10.3897/mycokeys.105.120534 (PMC11112159; doi:10.3897/mycokeys.105.120534)

Supplementary material 1

Supplementary data

Authors: Qi-Chao Guo, Shan Liu, Ya-Zhuo Qiao, Feng-Li Hui

Data type: pdf

Explanation note: Fig. S1. Maximum likelihood phylogenetic tree of *Teunia* generated from the ITS sequence data. The tree is rooted with *Cryptococcus amylolentus* CBS 6039^{T} and *Cryptococcus neoformans* CBS 8710^{T}. Bootstrap values (MLBS ≥ 50% and BPP ≥ 0.95) are displayed near branches. Type strain sequences are marked with (T). New species are highlighted in bold font.

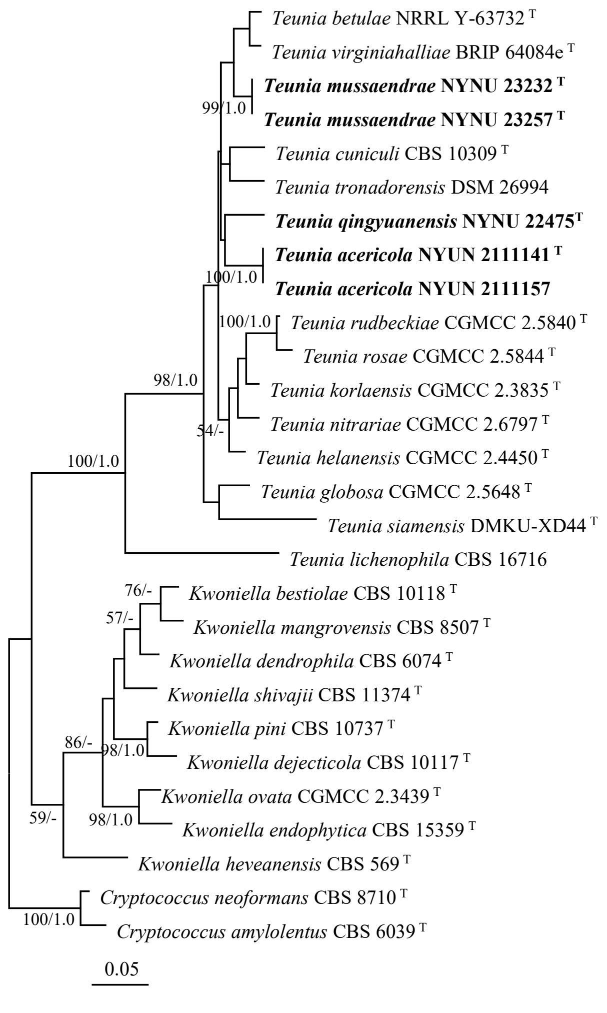

Supplement: Supplementary material 1 — Supplementary data [file mycokeys-105-139-s001.pdf]
